# A Nomogram for Predicting the Risk of Spinal Anesthesia-Induced Hypotension in Older Patients

**DOI:** 10.3390/diagnostics16040557

**Published:** 2026-02-13

**Authors:** Bingyi Wang, Zitian Chen, Qiaoyu Han, Yi Feng, Luyang Jiang, Bailin Jiang

**Affiliations:** 1 Department of Anesthesiology, Peking University People’s Hospital, Beijing 100044, China; wangby0501@163.com (B.W.); c986450629@163.com (Z.C.); hanqiaoyu1992@163.com (Q.H.); doc_yifeng@163.com (Y.F.); 2 Department of Anesthesiology, Beijing Chest Hospital, Capital Medical University, Beijing 101149, China

**Keywords:** elderly, spinal anesthesia, hypotension, echocardiography, nomogram, risk

## Abstract

**Background**: Hypotension is a common complication following spinal anesthesia, and it is particularly prevalent in older patients. The study aimed to develop and validate a nomogram integrating echocardiographic and clinical predictors for spinal anesthesia-induced hypotension (SAIH) in older patients. **Methods**: This was an observational cohort study conducted at Peking University People’s Hospital. A total of 865 older patients (age ≥ 65), enrolled from 1 January 2023 to 31 December 2024, were randomly split into a training set (70%) and an internal validation set (30%). For temporal external validation, 349 patients from January to March 2025 were enrolled. LASSO, univariable, and multivariate logistic regression analyses were used to identify predictive factors. A nomogram was subsequently developed based on the results of multivariate logistic regression, and its predictive efficacy was evaluated via both internal and temporal external validation. **Results**: SAIH occurred in 271 patients (44.8%) in the training set, 110 patients (42.3%) in the internal validation set, and 173 patients (49.6%) in the external validation set. Age, body mass index (BMI), bupivacaine dose, sensory block level, baseline systolic blood pressure (SBP), history of hypertension, interventricular septum thickness at end-diastole (IVSd), early diastolic mitral annular velocity (e’), and E/e’ ratio were significant predictors of SAIH on multivariate analysis. The diagnostic performance of the nomogram was favorable (AUC = 0.885, 95% CI: 0.859–0.911). The AUC values of the internal validation set and temporal external validation set were 0.856 (0.811–0.901) and 0.895 (0.863–0.927). **Conclusions**: This study identifies age, BMI, bupivacaine dose, sensory block level, baseline SBP, history of hypertension, and IVSd as predictors of SAIH with good discrimination and clinical utility. We present a predictive nomogram that accurately predicts SAIH in older patients. The external validation illustrates its generalizability.

## 1. Introduction

Spinal anesthesia (SA) provides favorable surgical conditions but carries significant hemodynamic risks, with an incidence of hypotension of 15–33%, representing the most common complication [1,2]. This phenomenon stems from SA-induced sympathetic blockade, which reduces systemic vascular resistance and venous return [3]. The elderly are particularly susceptible, with a >70% risk of hypotension due to age-related vascular stiffening and frequent comorbidities [4,5]. Uncontrolled spinal anesthesia-induced hypotension (SAIH) triggers acute hemodynamic instability and a cascade of severe perioperative complications in older patients: reduced coronary perfusion increases the risk of myocardial ischemia, arrhythmias, and even myocardial infarction in patients with underlying coronary artery disease; impaired cerebral blood flow autoregulation leads to cerebral hypoperfusion, presenting as dizziness, confusion, or ischemic stroke; and diminished renal perfusion precipitates acute kidney injury, particularly in those with pre-existing renal insufficiency. These adverse events collectively elevate perioperative mortality and morbidity, prolong hospital stays, and increase the risk of long-term cardiovascular and cerebrovascular sequelae [6,7,8,9].

Traditional methods for predicting spinal anesthesia-induced hypotension (SAIH) via preoperative assessment and intraoperative monitoring exhibit limited accuracy. Transthoracic echocardiography (TTE) has significantly improved the precision and safety of anesthetic management by enabling perioperative applications—preoperative risk assessment, real-time intraoperative monitoring, and postoperative complication diagnosis—which is particularly valuable for high-risk patients with hemodynamic instability [10]. Numerous studies have shown that preoperative echocardiographic parameters, including maximal inferior vena cava diameter (dIVCmax), inferior vena cava collapsibility index (IVCCI), and the dIVCmax-to-IVCCI ratio, are significant predictors of hypotension after SA in older patients [11,12,13].

Additional echocardiographic and clinical parameters may predict hypotension; however, the current lack of integrated models impedes accurate risk assessment. Nomograms serve as an efficient graphical method for multivariable risk prediction [14]. The present study aimed to develop and validate a nomogram integrating echocardiographic and clinical parameters to identify older patients at high risk of post-SA hypotension, thereby facilitating targeted prevention.

## 2. Materials and Methods

### 2.1. Study Design and Patient Enrollment

**Figure 1:**
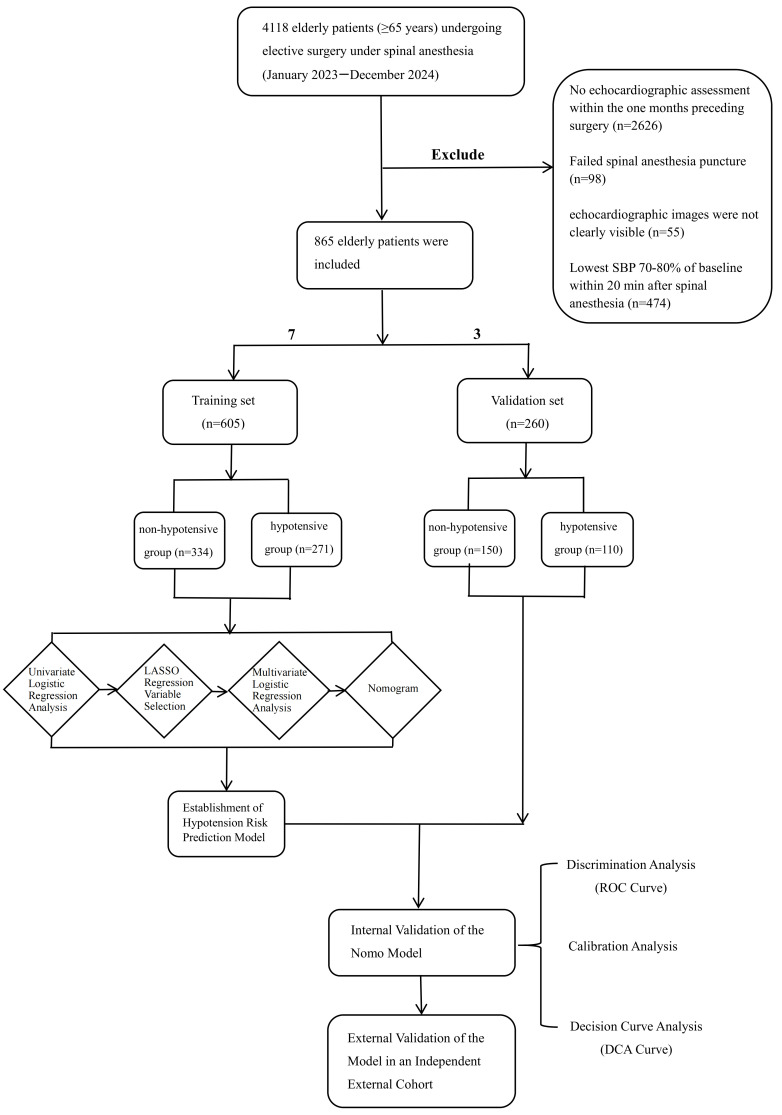
Flowchart of this study.

This was a retrospective cohort study, and reporting adheres to the STROBE guidelines. A total of 865 patients were enrolled from the clinical database of Peking University People’s Hospital between January 2023 and December 2024. The inclusion criteria were as follows: (1) age ≥ 65 years; (2) elective SA surgery; (3) preoperative echocardiographic examination within one month; and (4) American Society of Anesthesiologists Physical Status Classification System (ASA) I-III. The exclusion criteria were as follows: (1) failed SA puncture and (2) invisible echocardiographic images. The dataset was randomly split into training and validation sets at a 7:3 ratio. The training set was used for model development, while the validation set (150 non-hypotension and 110 hypotension cases) served for internal validation. For temporal external validation, 349 patients from January to March 2025 (176 non-hypotension and 173 hypotension cases) were enrolled. The flowchart of this study is shown in Figure 1.

### 2.2. Anesthesia Protocol and Hemodynamic Management

Prior to SA, midazolam was intravenously administered at a dose of 0.5–1 mg according to the patient’s individual conditions. Upon admission to the operating room, all patients received an intravenous infusion of lactated Ringer’s solution at a rate of 3–5 mL·kg^−1^ ·h^−1^ before spinal puncture. The infusion rate was individualized to prevent volume overload induced by rapid fluid administration. Preoperative unilateral nerve blocks were not administered for orthopedic surgeries included in this study. Standardized SA was performed in the lateral decubitus position at the L2–L3 or L3–L4 interspace using hypobaric bupivacaine (0.3%, 3–4 mL, 9–12 mg) via a 25-gauge Quincke needle, with the dose individually adjusted according to patient characteristics, surgical requirements, and clinical practice guidelines. The sensory block level was assessed via pinprick testing at 20 min after SA and recorded as the highest dermatomal level of analgesia, expressed as a vertebral segment. Perioperative antihypertensive management included discontinuing Angiotensin-Converting Enzyme Inhibitors (ACEIs) and Angiotensin II Receptor Blockers (ARBs), while continuing calcium channel blockers and β-blockers. Blood pressure (BP) was monitored noninvasively at 3 min intervals using an automated oscillometric device (PHILIPS MP70) on the brachial artery. Baseline BP was defined as the first measurement obtained after midazolam premedication. Hypotension was strictly defined as a ≥30% reduction in systolic blood pressure (SBP) from baseline within 20 min following SA administration [15]. Mean arterial pressure (MAP) was calculated as diastolic blood pressure (DBP) + (SBP−DBP)/3. A protocolized intervention was initiated if hypotension persisted for >3 min: phenylephrine 50 μg or ephedrine 6 mg for hypotension, and atropine 0.5 mg for bradycardia (heart rate (HR) < 50 bpm). Patients with SBP reductions of 20–30% from baseline were excluded.

### 2.3. Echocardiography

All transthoracic echocardiographic examinations were performed within one month before surgery in the Department of Echocardiography of our hospital by certified cardiac sonographers using a standardized protocol with an M5Sc 2–5 MHz phased-array transducer (VIVID E95 R4 WXB, Mindray, Beijing, China). From the parasternal long-axis view, M-mode measurements included left atrial diameter (LAD), interventricular septum thickness at end-diastole (IVSd), left ventricular end-systolic diameter (LVESD), left ventricular end-diastolic diameter (LVEDD), left ventricular posterior wall thickness at end-diastole (LVPWD), left ventricular mass (LVM), left ventricular end-diastolic volume (LVEDV), and left ventricular end-systolic volume (LVESV). Left ventricular ejection fraction (LVEF) was calculated using the Teichholz method in patients with normal wall motion or the modified biplane Simpson’s method in those with segmental wall motion abnormalities. Pulsed-wave spectral Doppler was used to measure Mitral early diastolic peak velocity (E) and mitral late diastolic peak velocity (A) in the apical four-chamber view. Tissue Doppler imaging (TDI) with pulsed-wave was used to measure early diastolic mitral annular velocity (e’) at the septal annulus in the apical four-chamber view, and the E/A ratio (E/A) and E/e’ ratio were calculated.

### 2.4. Data Collection

Patient data were collected from the clinical electronic medical record system by two authors (Z.T.C. and B.Y.W.), with duplicate checks performed for the data. Collected data included patients’ basic clinical data (sex, age, height, weight, body mass index (BMI), type of surgery), medical history (hypertension, diabetes mellitus, coronary artery disease), anesthetic data (ASA, fasting duration (calculated from 10:00 p.m. on the preoperative day to the time of operating room admission), preoperative fluid administration (intravenous infusion of glucose-saline solution at 125 mL/h, initiated at 10:00 a.m. on the operative day), dose of bupivacaine, sensory block level, baseline and post-SA SBP, DBP, MAP, and HR).

### 2.5. Statistical Analysis

Statistical analysis was conducted using R 4.4.2. The sample size was calculated using the 10 events per variable (10 EPV) rule. Patients with missing echocardiographic parameters (missing rate > 20%) were excluded, and the remaining missing data were imputed via multiple imputation. The normality of data was assessed using the Shapiro–Wilk test. Continuous variables are reported as mean ± standard deviation (SD) and compared using the Student’s t-test for independent samples, while non-normally distributed continuous variables are presented as median (interquartile range, IQR) and analyzed using the Mann–Whitney U test. Categorical variables are presented as counts and percentages and compared using the chi-square test or Fisher’s exact test (if an expected value of ≤5 was observed). A *p* value < 0.05 was considered statistically significant. Univariate logistic regression was first performed on the training dataset to identify potential predictors (*p* < 0.1). Significant variables were then included in Least Absolute Shrinkage and Selection Operator (LASSO) logistic regression for feature selection. Subsequently, the selected variables were entered into multivariate logistic regression, and predictors with statistical significance (*p* < 0.05) were retained in the final model. The final prediction model was presented as a nomogram for clinical use. Receiver operating characteristic (ROC) curves were constructed and the area under the curve (AUC) was calculated to assess the predictive performance of the model. The agreement between predicted probabilities from the nomogram and actual probabilities was assessed using calibration curves. Decision curve analysis (DCA) was performed to examine the clinical applicability of the prediction model in practical decision-making.

## 3. Results

### 3.1. Patient Demographic and Clinical Characteristics

SAIH occurred in 271 patients (44.8%) in the training set, 110 patients (42.3%) in the internal validation set, and 173 patients (49.6%) in the external validation set. The demographic and clinical characteristics of the training set, internal validation set, and external validation set are detailed in Table 1. There were no statistically significant differences between the training and internal validation sets in terms of SAIH occurrence, basic clinical data, medical history, anesthetic data, and echocardiographic parameters (*p* > 0.05) (Table S1).

### 3.2. Univariate Analysis of Hypotensive vs. Non-Hypotensive Groups in the Training Set

In the training set, compared with the non-hypotensive group, the hypotensive group had higher age, weight, and BMI but lower height in basic clinical characteristics; a higher prevalence of hypertension in medical history; a higher dose of bupivacaine, baseline SBP, baseline MAP, and sensory block level but lower post-SA SBP, DBP, and MAP in anesthetic parameters; and higher LAD, IVSd, LVM, E, and E/e’ but lower A and e’ in echocardiographic parameters (*p* < 0.1) (Table 1).

### 3.3. LASSO Regression Analysis with 10-Fold Cross-Validation

**Figure 2:**
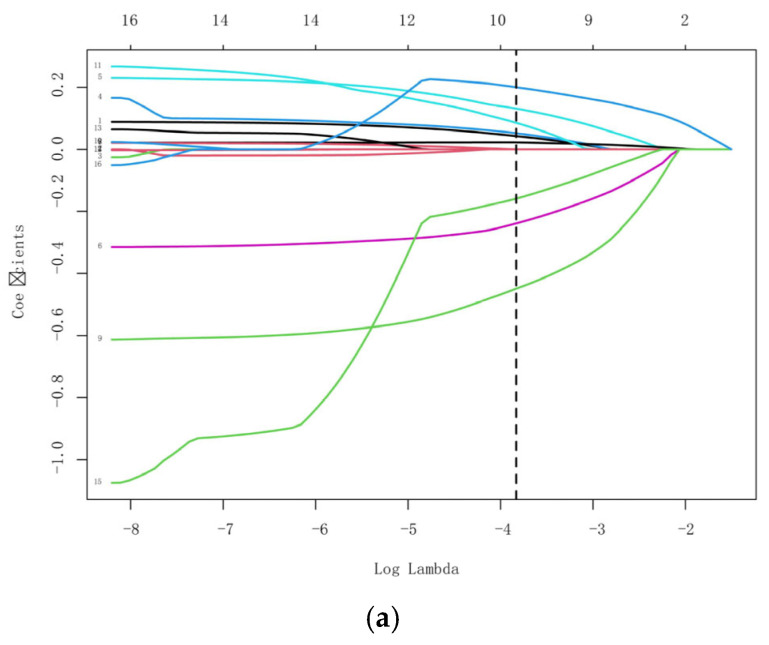

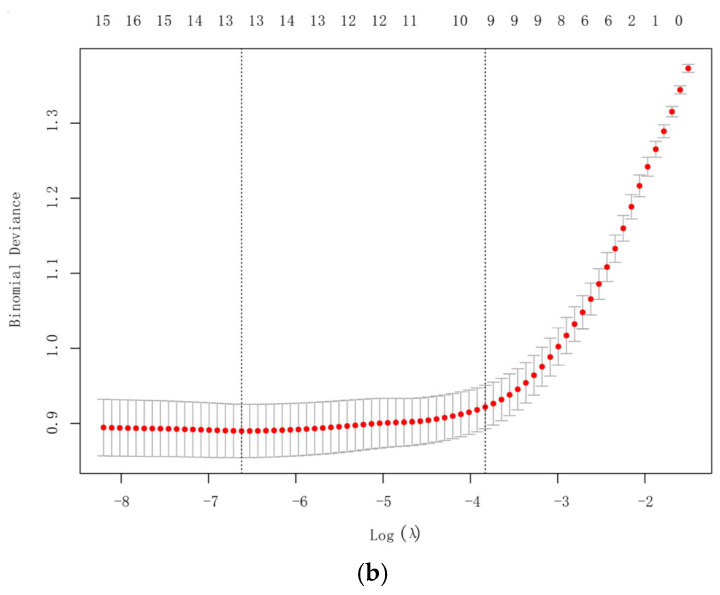
Feature selection using LASSO regression. (**a**). LASSO coefficient profiles of 16 clinical features. The plot was created using a logarithmic scale for the lambda values. A vertical line was added to indicate the lambda value selected through tenfold cross-validation. This optimal lambda value led to the identification of nine features with nonzero coefficients. (**b**). The optimal parameter λ selection in the LASSO model employed tenfold cross-validation using a minimum criterion approach. The optimal values of λ are represented by dotted vertical lines. Among these values, λ = 0.022 was selected as the optimal choice.

A total of 16 variables with *p* < 0.1 in univariate analysis (age, height, weight, BMI, bupivacaine dose, sensory block level, baseline SBP, baseline MAP, history of hypertension, LAD, IVSd, LVM, E, A, e’, E/e’) were entered into LASSO regression with 10-fold cross-validation. Nine predictors with nonzero coefficients (age, BMI, bupivacaine dose, sensory block level, baseline SBP, history of hypertension, IVSd, e’, E/e’) were included in multivariate analysis (Figure 2).

### 3.4. Multivariable Logistic Regression Analysis for Identifying Independent Predictors

The results indicated that age (OR: 1.083, 95% CI: 1.042–1.127), BMI (OR: 1.104, 95% CI: 1.031–1.182), dose of bupivacaine (OR: 1.216, 95% CI: 1.092–1.354), baseline SBP (OR: 1.031, 95% CI: 1.015–1.047), history of hypertension (yes) (OR: 1.78, 95% CI: 1.13–2.82), IVSd (OR: 1.210, 95% CI: 1.013, 1.444), and E/e’ (OR: 1.289, 95% CI: 1.182–1.405) are risk factors for the occurrence of hypotension, while sensory block level (OR: 0.709, 95% CI: 0.594–0.846) and e’ (OR: 0.761, 95% CI: 0.630–0.920) are protective factors against hypotension, with statistical significance (*p* < 0.05) (Table 2).

### 3.5. Development of a Nomogram Model for Spinal Anesthesia-Induced Hypotension in Older Patients

**Figure 3:**
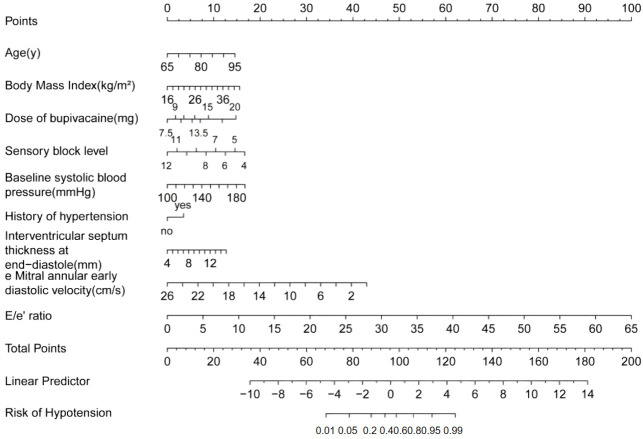
Nomogram for predicting SAIH in older patients.

A nomogram model was constructed based on the results of multivariate logistic regression, including the following predictors: age, BMI, dose of bupivacaine, sensory block level, baseline SBP, history of hypertension, IVSd, e’, and E/e’ (Figure 3). The results of the multivariate logistic regression analysis for the diagnostic outcome are presented, with the derived linear predictor equation as follows: Logit (P) = −16.169 + 0.080 × age + 0.099 × BMI + 0.195 × dose of bupivacaine − 0.344 × sensory block level + 0.031 × baseline SBP + 0.579 × history of hypertension + 0.190 × IVSd − 0.273 × e’+ 0.254 × E/e’. For patients undergoing SA, anesthesiologists should extract each clinical indicator shown in the nomogram sequentially. To use the nomogram in clinical practice, a vertical line can be drawn upward directly from the value of each variable to the scoring line to obtain the corresponding score. The total score is calculated by summing the scores of all variables, and a vertical line is then drawn downward from the total score to the “Risk of Hypotension” axis to directly read the SAIH probability for each patient. Anesthesiologists can quickly assess the risk of SAIH in patients before surgery using this nomogram. For high-risk patients, preventive interventions can be implemented in advance, such as adjusting the bupivacaine dose, optimizing volume management, and prophylactically administering vasoactive drugs.

### 3.6. Online Dynamic Nomogram Establishment

**Figure 4:**
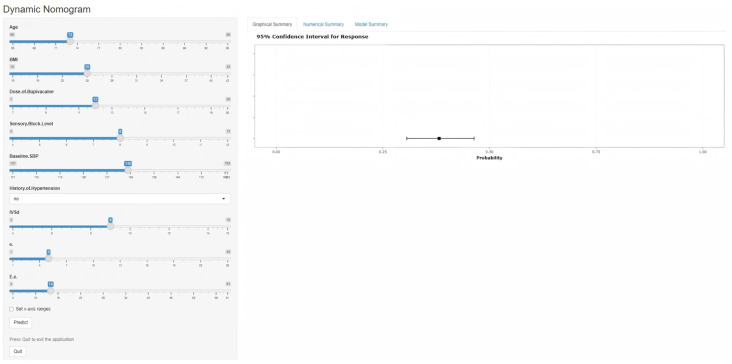
Web-based dynamic nomogram.

Based on the predictors screened by the multivariate logistic regression analysis and the graphical nomogram model, an online dynamic nomogram was then established (website: https://modelforsaihinelderly.shinyapps.io/dynnomapp/ accessed on 2 February 2026). Clinicians can easily input the relevant clinical and echocardiographic parameters of individual patients and quickly obtain the personalized SAIH risk probability. A screenshot of the online prediction tool is provided in Figure 4.

### 3.7. Model Validation

**Figure 5:**
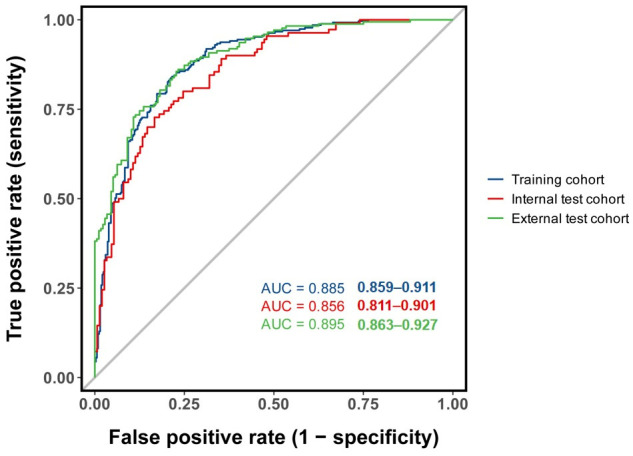
Discriminative performance of the nomogram. Training set (blue line), AUC: 0.885, 95% CI: (0.859–0.911); internal validation (red line), AUC: 0.856, 95%, CI: (0.811–0.901); external validation (green line), AUC: 0.895, 95% CI: (0.863–0.927).

**Figure 6:**
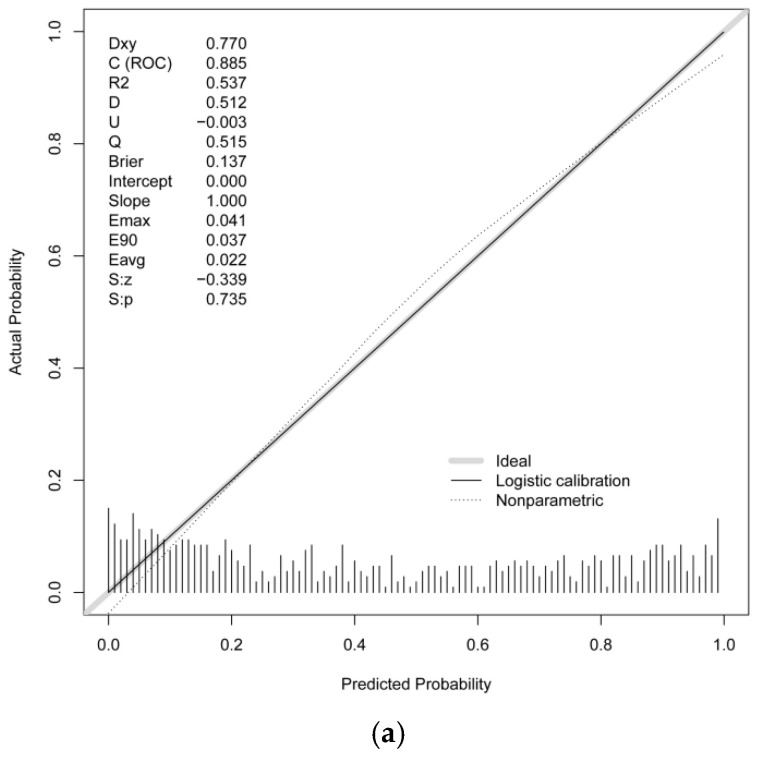

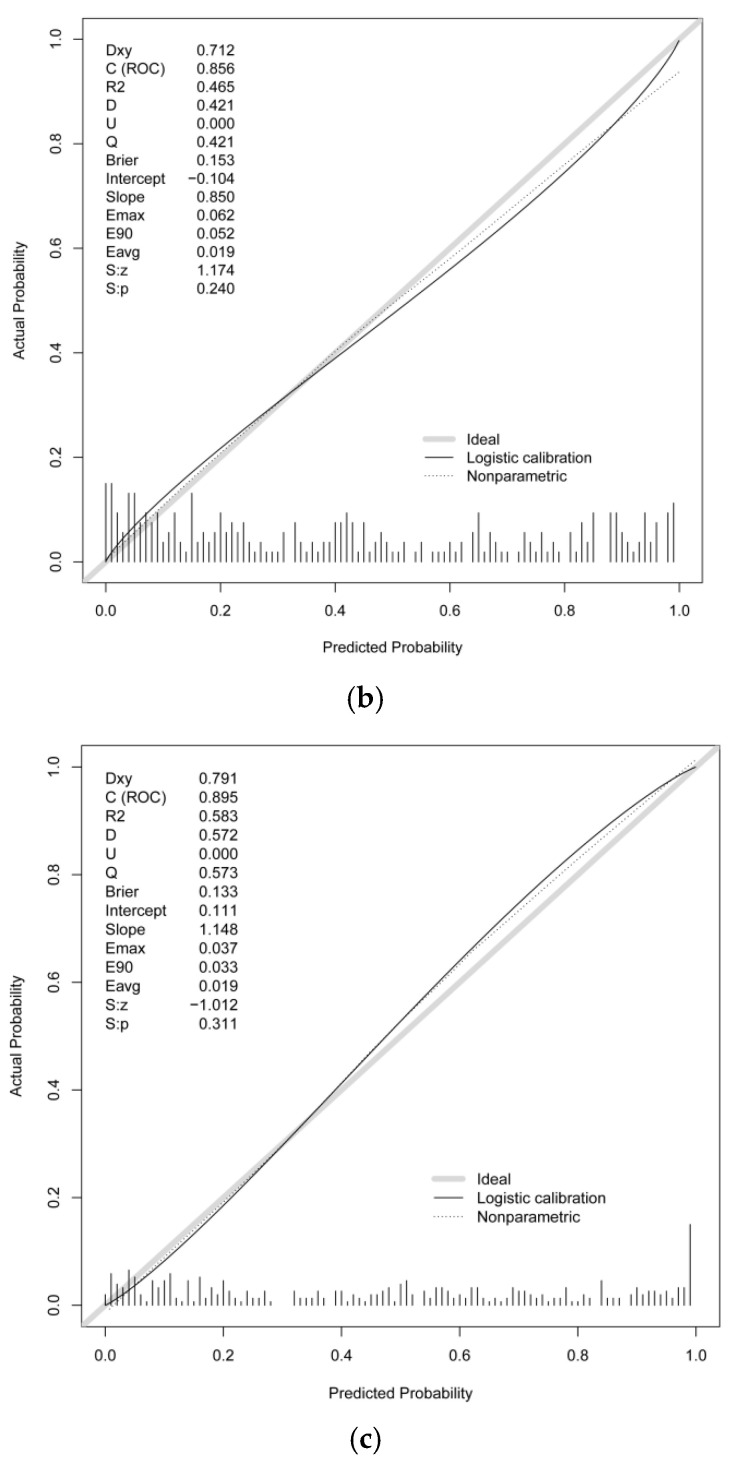
Calibration analysis of the nomogram. (**a**) Training set; (**b**) internal test set; (**c**) external test set.

**Figure 7:**
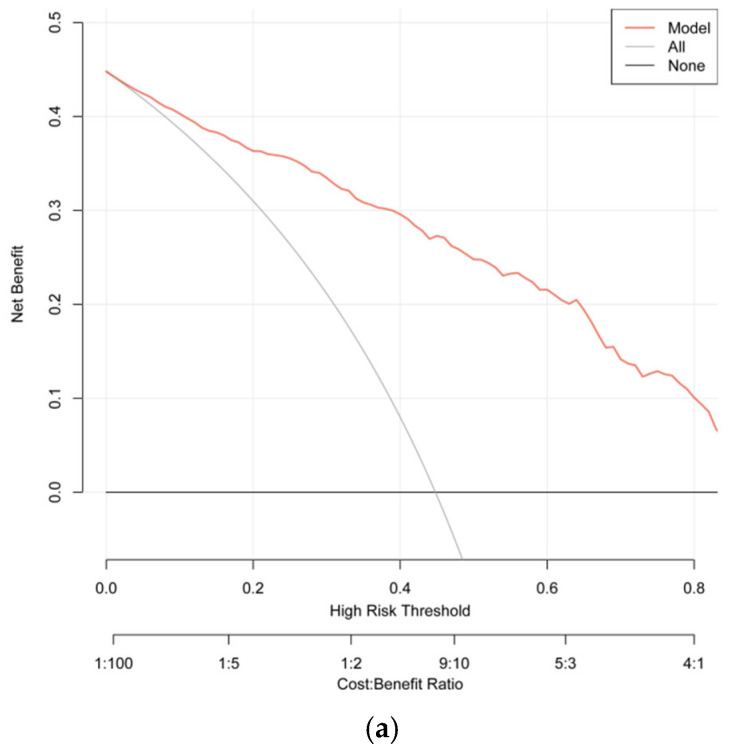

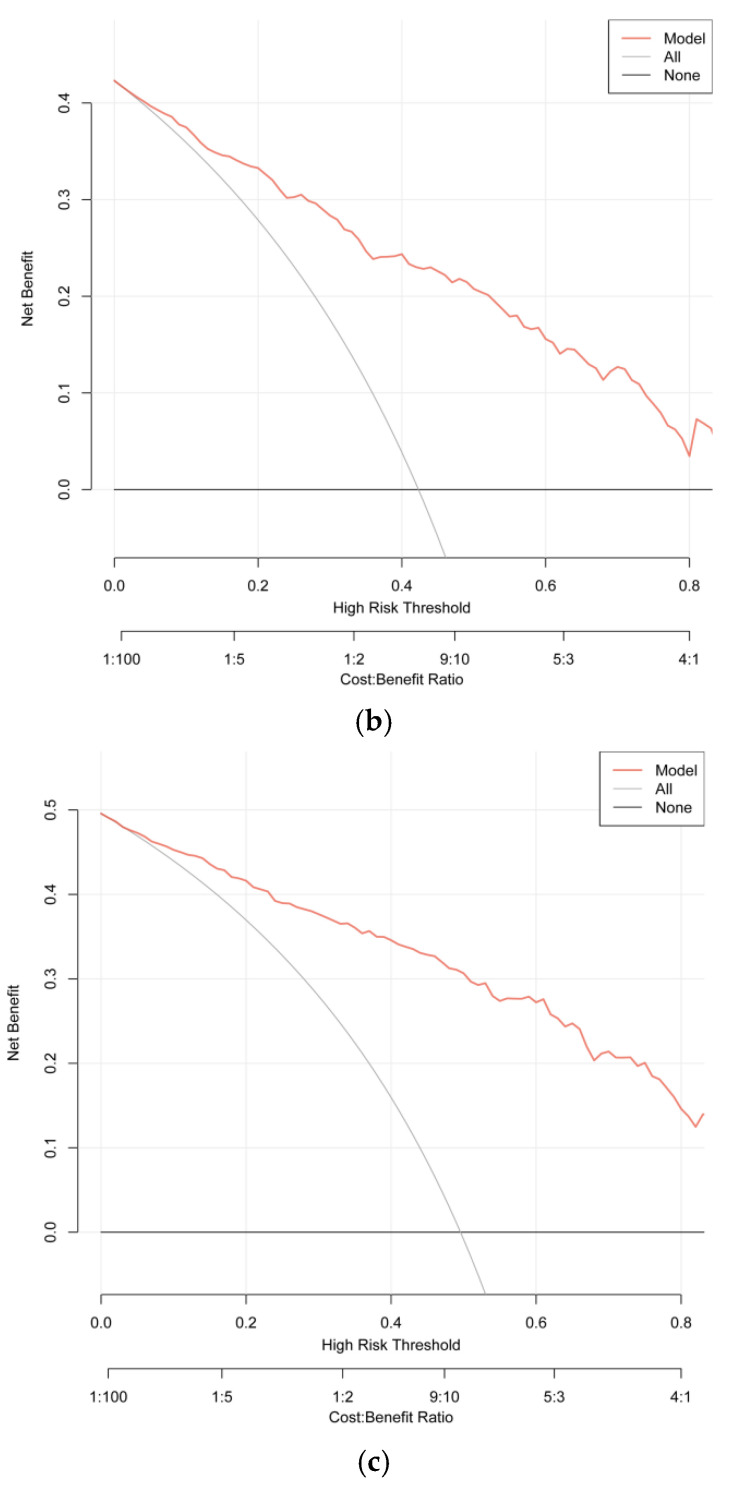
Clinical utility assessment by decision curve analysis. (**a**) Training set; (**b**) Internal test set; (**c**) External test set. Clinical model (red line), total (gray line, indicating assumption of hypotension in all patients), and none (horizontal solid line, black line, indicating assumption of hypotension in no patients).

The AUC values were 0.885 (95% CI: 0.859–0.911) for the training set (cutoff: 0.395, sensitivity: 0.841, specificity: 0.784), 0.856 (95% CI: 0.811–0.901) for the internal validation set (cutoff: 0.481, sensitivity: 0.833, specificity: 0.727), and 0.895 (95% CI: 0.863–0.927) for the external validation set (cutoff: 0.560, sensitivity: 0.861, specificity: 0.767) (Figure 5). Additionally, the Nagelkerke R^2^ value of the final model was 0.5373, indicating that the nine selected predictors can explain 53.73% of the variance in SAIH incidence, reflecting a moderate-to-strong explanatory power of the model. Calibration curves showed good consistency between predicted and observed probabilities, supported by Hosmer–Lemeshow test results (training set: χ^2^ = 12.755, *p* = 0.121; internal validation set: χ^2^ = 9.395, *p* = 0.310; external validation set: χ^2^ = 2.432, *p* = 0.965) and favorable Brier scores (0.137, 0.153, and 0.133, respectively) (Figure 6). Decision curve analysis (DCA) showed that the standardized net benefit of the prediction model was significantly higher than that of both the “treat-all” and “treat-none” strategies (Figure 7).

## 4. Discussion

The present study developed a predictive model for SAIH in elderly patients, which exhibits good discriminative ability. Multivariable analysis identified nine independent predictors: age, BMI, dose of bupivacaine, sensory block level, baseline SBP, history of hypertension, IVSd, e’, and E/e’.

Previous studies have reported that the incidence of SAIH in elderly patients is 70% [5]. In contrast, the incidence of SAIH in our training set was significantly lower at 44.8%. This discrepancy may be attributed to the following reasons: (1) While prior studies defined hypotension as a >20% reduction in SBP from baseline [16,17], we adopted a stricter criterion of ≥30%, considering that older patients often have higher baseline SBP due to arterial stiffness, reduced vascular compliance, and lifestyle factors [18,19]. This stricter threshold enhances diagnostic specificity by minimizing the misclassification of physiological BP fluctuations and avoiding unnecessary interventions [20]. (2) We defined the elderly as ≥65 years [21], whereas earlier studies used a cutoff of ≥75 years [22]. Since the risk of hypotension increases with age, the inclusion of younger elderly patients may have contributed to the lower observed incidence. Our results confirmed known predictors such as age, history of hypertension history, BMI, sensory block level, and the dose of bupivacaine [5,23,24,25], while also identifying novel predictors, including baseline SBP, IVSd, e′, and E/e′.

The cardiovascular system undergoes substantial structural and functional changes with aging [26]. In older people, increased vascular stiffness, decreased adrenergic receptor responsiveness, and impaired autonomic reflexes contribute to both diastolic dysfunction and reduced myocardial contractility [27]. These changes diminish the compensatory response of cardiac output (CO) during hypoperfusion and impair the autoregulatory capacity to maintain systemic vascular resistance (SVR) [21], thereby increasing the risk of post-SA hypotension.

Obesity is associated with increased intra-abdominal pressure, which reduces cerebrospinal fluid (CSF) volume and weakens the supportive and regulatory effects of CSF on surrounding relevant structures. This, in turn, may compress the intervertebral foramina and inferior vena cava. Such compression further reduces CSF volume, potentially increasing the concentration of local anesthetics and promoting cephalad spread. However, the spread of local anesthetics is not linearly associated with CSF volume, and individual variability along with compensatory hemodynamic mechanisms contributes to heterogeneous responses [28,29,30]. This heterogeneity may be due to differences in study populations, anesthetic protocols, and the definition or monitoring of hypotension. Obesity may affect SA outcomes through multiple pathophysiological pathways involving the respiratory, cardiovascular, and endocrine systems [31].

There is a clear dose-dependent relationship between the administration of local anesthetics and the incidence of SAIH [32]. The core mechanism of hypotension following spinal anesthesia is vasodilation induced by sympathetic blockade, which subsequently reduces venous return [33]. Higher intrathecal doses of local anesthetics result in higher block levels, leading to more extensive sympathetic blockade and increasing the risk of SAIH [34]. Dose-reduction strategies have been consistently shown to reduce this risk [35,36]. Combining low-dose local anesthetics with opioids enhances analgesic efficacy while maintaining cardiovascular stability and facilitating faster recovery [37,38].

Higher sensory block levels are a major cause of SAIH, as they induce vasodilation and decrease SVR [32]. Sympathetic nerve fibers regulating vascular tone originate from T5–L1, and cardiac accelerator fibers arise from T1–T4 [39,40]. Thus, higher block levels result in more extensive sympathetic blockade and an increased risk of SAIH [41,42]. Several studies have reported an increased risk of hypotension at block levels of T5 or above [23,43]. Seltenrich et al. reported that different rates of sympathetic blockade may affect the incidence of hypotension, even when the final block levels are similar [44]. Therefore, both the block level and rate of ascent should be closely monitored to reduce the risk of SAIH.

Our study found that elevated preoperative SBP is an independent risk factor for SAIH. Patients with higher baseline BP may have elevated sympathetic activity and thus experience a more profound sympatholytic effect following SA, increasing their susceptibility to SAIH. This finding is consistent with that of Prashanth et al. [45], who reported that patients with increased preoperative sympathetic activity were more prone to hypotension.

Essential hypertension is more prevalent in the elderly due to arterial stiffness and endothelial dysfunction [46]. Chronic hypertension induces vascular wall thickening and reduced compliance, increasing the risk of SAIH. Racle et al. [47] reported that hypertensive patients have twice the risk of post-SA hypotension. Antihypertensive medications also influence this risk. Kaimar et al. [48] reported no significant difference in the incidence of SAIH between patients receiving β-blockers and those receiving calcium channel blockers, whereas Coriat et al. [49] reported that continuing ACEI on the day of surgery increased the risk of hypotension. Thus, both hypertension and its pharmacologic management significantly impact perioperative hemodynamic stability.

TTE has emerged as a crucial noninvasive bedside tool for the rapid assessment of cardiac function and volume status in the operating room [50]. Although left ventricular diastolic dysfunction (LVDD) affects 30% of the general population, its prevalence increases to 65–86% in the elderly [51,52]. Patients with LVDD are often asymptomatic at rest, with abnormalities only detected under stress [53]. Echocardiographic markers such as left atrial area, tricuspid regurgitant velocity, E/e’, e’, E, and E/A ratio are established indicators of LVDD [13,54,55]. e′ reflects early myocardial relaxation, and reduced values indicate diastolic dysfunction. In contrast to E, which is highly influenced by preload, e’ is a less preload-dependent parameter. E/e’ is an indicator that fully accounts for preload effects and is normalized by e’. Diastolic dysfunction leads to insufficient ventricular filling during diastole and a reduction in ventricular blood reserve; due to this inadequate diastolic filling, stroke volume decreases, and even with a compensatory increase in heart rate, cardiac output remains reduced. While SA-induced reduction in venous return contributes to SAIH, it is not the sole cause; decreased ventricular compliance leads to diastolic impairment, which ultimately restricts ventricular filling and reduces stroke volume. E is highly preload-dependent and primarily reflects instantaneous preload status, making it inadequate for the comprehensive assessment of these complex diastolic abnormalities. In contrast, e’ and E/e’ are less influenced by preload and better reflect intrinsic myocardial properties, thereby demonstrating superior predictive value for SAIH.

Our study newly identified IVSd as a potential predictor. The normal range of IVSd is approximately 6–12 mm, which can reflect the degree of myocardial hypertrophy and diastolic function status. IVSd is often elevated in patients with hypertension and hypertrophic cardiomyopathy [56]. Older patients may experience myocardial remodeling due to long-term hypertension, coronary heart disease, and other conditions, leading to IVSd thickening. IVSd thickening is associated with a decrease in ventricular diastolic compliance, which in turn results in diastolic dysfunction. In older patients with IVSd thickening and diastolic dysfunction, the left ventricular end-diastolic volume is reduced, and blood reserve is decreased. After SA, peripheral vasodilation further reduces venous return. Owing to the reduced ventricular volume and left ventricular outflow tract obstruction, the heart exhibits a decrease in stroke volume. Compared with normal IVSd, interventricular septal hypertrophy increases the incidence of coronary heart disease and myocardial infarction [57]. Nevertheless, further validation is needed to clarify its role in predicting SAIH.

Notably, the Nagelkerke R^2^ value of the final model was 0.5373, demonstrating that the nine identified predictors could collectively explain 53.73% of the variance in SAIH incidence. This moderate-to-strong explanatory power, in combination with the favorable discriminative ability (AUC = 0.885 in the training set and 0.895 in the external validation set) and favorable calibration performance, confirms that our model has robust statistical reliability and high clinical applicability. This superiority can be ascribed to the integration of echocardiographic parameters associated with left ventricular diastolic function, which effectively compensates for the inherent limitations of conventional models relying exclusively on clinical variables.

This study aimed to construct a nomogram to facilitate early identification of older patients at risk for SAIH. Current prevention strategies include fluid preloading and prophylactic vasopressor use [33]. Other effective measures involve correcting hypovolemia, limiting block height, using the Trendelenburg position to improve venous return, and employing cautious sedation [58]. However, several limitations of this study should be noted: (1) The time window for echocardiographic parameters involved in this study was excessively long. These parameters were not collected on the day before surgery or immediately before anesthesia, which fails to accurately reflect the patient’s physiological state during surgery. (2) Conventional echocardiographic parameters do not include indicators for evaluating volume status; thus, volume status indicators like IVCd and IVCCI were not incorporated into the model, failing to account for fasting-induced volume depletion. Moreover, this study lacks the inclusion of echocardiographic parameters related to cardiac valve function, such as the orifice area of the aortic valve and mitral valve, peak transvalvular flow velocity, and mean transvalvular pressure gradient. Aortic valve or mitral valve stenosis can seriously affect the hemodynamic stability of older patients, which may further impact the occurrence of SAIH. Future studies could expand the range of echocardiographic parameters and incorporate biomarkers such as inflammatory or myocardial injury markers. (3) The model exhibited excessively high performance in both internal and external validation sets, suggesting a potential overfitting issue; additionally, the single-center design may introduce selection bias and limit the generalizability of the findings. Therefore, further validation using multi-center external validation sets is required.

## 5. Conclusions

This study developed a predictive model integrating clinical and echocardiographic parameters for SAIH in older patients. Key independent predictors identified include age, BMI, bupivacaine dose, sensory block level, baseline SBP, history of hypertension, IVSd, e’, and E/e’. The model exhibits favorable predictive performance for clinical risk stratification and optimization of preventive strategies.

## Figures and Tables

**Table 1:** Baseline characteristics of the training, internal validation, and external test sets.

Characteristic	Training Set, N = 605			Internal Test Set, N = 260			External Test Set, N = 349		
	Control N = 334	Hypotension N = 271	*p*	Control N = 150	Hypotension N = 110	*p*	Control N = 176	Hypotension N = 173	*p*
Sex			0.726			0.070			0.481
Male	104 (31.14%)	88 (32.47%)		48 (32.00%)	24 (21.82%)		43 (24.43%)	48 (27.75%)	
Female	230 (68.86%)	183 (67.53%)		102 (68.00%)	86 (78.18%)		133 (75.57%)	125 (72.25%)	
Age(y)	72.00 (68.00, 75.00)	73.00 (69.00, 78.00)	<0.001	72.00 (69.00, 77.00)	72.00 (68.00, 78.00)	0.604	71.00 (67.00, 74.00)	73.00 (69.00, 79.00)	<0.001
ASA Physical Status Classification			0.192			0.231			0.005
II	243 (72.75%)	184 (67.90%)		106 (70.67%)	70 (63.64%)		127 (72.16%)	100 (57.80%)	
III	91 (27.25%)	87 (32.10%)		44 (29.33%)	40 (36.36%)		49 (27.84%)	73 (42.20%)	
Height (cm)	162.00 (158.00, 168.00)	160.00 (156.00, 166.00)	0.041	161.00 (158.00, 168.00)	158.50 (155.00, 164.00)	0.008	160.00 (156.00, 167.00)	161.00 (157.00, 167.00)	0.498
Weight (kg)	65.00 (58.00, 72.00)	68.00 (60.00, 75.00)	0.013	67.00 (60.00, 71.00)	65.00 (58.50, 75.00)	0.617	60.00 (55.00, 65.00)	66.00 (60.00, 73.00)	<0.001
Body Mass Index (kg/m^2^)	24.77 (22.86, 27.44)	25.85 (23.34, 28.65)	<0.001	25.04 (23.34, 27.34)	25.35 (22.77, 28.40)	0.488	23.23 (22.04, 24.03)	25.39 (23.80, 27.34)	<0.001
Type of Surgery			0.549			0.961			0.969
Orthopedics	228 (68.26%)	196 (72.32%)		110 (73.33%)	82 (74.55%)		139 (78.98%)	136 (78.61%)	
Gynecology	44 (13.17%)	32 (11.81%)		22 (14.67%)	16 (14.55%)		16 (9.09%)	15 (8.67%)	
Urology	62 (18.56%)	43 (15.87%)		18 (12.00%)	12 (10.91%)		21 (11.93%)	22 (12.72%)	
Fasting Duration (h)	13.77 (11.72, 15.35)	12.18 (9.50, 17.27)	0.108	13.64 (11.77, 15.43)	12.31 (9.50, 17.18)	0.332	13.43 (11.28, 16.03)	12.40 (9.50, 15.35)	0.031
Preoperative Fluid Administration (mL)	220.83 (0.00, 418.75)	22.92 (0.00, 658.75)	0.221	205.21 (0.00, 429.17)	38.54 (0.00, 647.50)	0.343	179.17 (0.00, 503.12)	50.00 (0.00, 418.75)	0.048
Dose of Bupivacaine (mg)	12.00 (10.50, 13.50)	13.50 (12.00, 15.00)	<0.001	12.00 (10.00, 13.50)	12.50 (10.50, 15.00)	<0.001	10.50 (10.50, 13.50)	12.00 (12.00, 15.00)	<0.001
Sensory Block Level	8.00 (8.00, 10.00)	8.00 (6.00, 8.00)	<0.001	8.00 (8.00, 10.00)	8.00 (6.00, 8.00)	<0.001	8.00 (8.00, 10.00)	8.00 (6.00, 8.00)	<0.001
Baseline Systolic Blood Pressure (mmHg)	141.50 (130.00, 151.00)	151.00 (140.00, 163.00)	<0.001	139.50 (128.00, 150.00)	150.00 (140.00, 162.00)	<0.001	138.50 (127.00, 151.00)	150.00 (141.00, 161.00)	<0.001
Baseline Diastolic Blood Pressure (mmHg)	74.95 ± 10.133	75.74 ± 10.466	0.348	74.53 ± 10.142	76.90 ± 10.451	0.069	75.13 ± 9.597	74.94 ± 10.006	0.858
Baseline Mean Arterial Pressure (mmHg)	96.83 ± 9.352	100.95 ± 9.620	<0.001	96.17 ± 8.931	101.35 ± 9.714	<0.001	96.44 ± 9.438	99.76 ± 7.827	<0.001
Baseline Heart Rate (bpm)	73.00 (66.00, 81.00)	75.00 (67.00, 82.00)	0.164	71.00 (65.00, 78.00)	76.00 (70.00, 85.00)	<0.001	71.50 (65.00, 78.00)	74.00 (66.00, 82.00)	0.023
History of Hypertension	128 (38.32%)	189 (69.74%)	<0.001	60 (40.00%)	74 (67.27%)	<0.001	99 (56.25%)	118 (68.21%)	0.021
History of Diabetes Mellitus	71 (21.26%)	64 (23.62%)	0.488	28 (18.67%)	23 (20.91%)	0.653	41 (23.30%)	55 (31.79%)	0.076
History of Coronary artery Disease	44 (13.17%)	43 (15.87%)	0.348	16 (10.67%)	14 (12.73%)	0.607	29 (16.48%)	28 (16.18%)	0.941
Left Atrial Anterior Posterior Diameter (cm)	3.50 (3.30, 3.90)	3.60 (3.40, 4.00)	0.008	3.60 (3.30, 3.90)	3.65 (3.30, 4.00)	0.391	3.60 (3.30, 3.90)	3.70 (3.40, 4.00)	0.006
Interventricular Septum Thickness at End-Diastole (mm)	8.70 (7.90, 9.60)	9.00 (8.10, 10.00)	0.005	8.70 (7.90, 9.70)	8.60 (8.10, 9.50)	0.510	8.90 (8.10, 9.50)	9.70 (8.50, 11.00)	<0.001
Left Ventricular End-Diastolic Diameter (cm)	4.80 (4.50, 5.10)	4.80 (4.50, 5.10)	0.228	4.70 (4.50, 5.00)	4.75 (4.50, 5.00)	0.614	4.80 (4.50, 5.10)	4.70 (4.40, 5.00)	0.075
Left Ventricular End-Systolic Diameter (cm)	2.90 (2.70, 3.20)	3.00 (2.80, 3.20)	0.333	2.95 (2.70, 3.20)	2.90 (2.70, 3.20)	0.411	3.00 (2.80, 3.20)	2.90 (2.70, 3.10)	0.006
Left Ventricular Posterior Wall Thickness at End-Diastole (cm)	0.87 (0.78, 0.94)	0.87 (0.80, 0.95)	0.154	0.87 (0.80, 0.95)	0.87 (0.80, 0.94)	0.559	0.87 (0.80, 0.94)	0.87 (0.80, 0.94)	0.840
Left Ventricular Mass (g)	140.55 (117.60, 161.00)	146.30 (125.40, 168.40)	0.013	139.85 (121.70, 163.90)	138.30 (123.30, 155.50)	0.991	142.50 (118.95, 164.20)	138.60 (117.60, 158.30)	0.212
Left Ventricular Ejection Fraction (%)	67.84 (63.68, 72.22)	67.83 (64.08, 71.27)	0.820	68.09 (63.77, 72.10)	69.18 (65.26, 71.89)	0.248	66.62 (62.81, 69.43)	67.81 (63.46, 72.15)	0.026
Left Ventricular End-Diastolic Volume (mL)	105.75 (91.70, 122.00)	108.80 (94.10, 122.00)	0.262	103.95 (91.40, 120.30)	104.35 (91.40, 118.10)	0.697	107.90 (91.40, 122.06)	102.20 (88.10, 116.20)	0.070
Left Ventricular End-Systolic Volume (mL)	33.45 (27.20, 39.70)	33.70 (29.00, 39.70)	0.370	33.55 (27.00, 40.10)	31.70 (26.20, 39.70)	0.389	34.60 (30.55, 39.70)	33.00 (27.20, 38.30)	0.008
Mitral Early Diastolic Peak Velocity (cm/s)	64.70 (54.60, 77.50)	69.90 (58.30, 82.10)	0.003	65.60 (54.60, 82.60)	65.30 (57.50, 78.30)	0.566	67.50 (58.60, 78.80)	71.30 (56.20, 86.90)	0.126
Mitral Late Diastolic Peak Velocity (cm/s)	92.50 (83.70, 104.70)	97.10 (84.00, 111.00)	0.007	92.45 (82.10, 103.10)	97.25 (86.50, 110.90)	0.014	95.30 (85.00, 103.25)	102.00 (87.80, 112.70)	0.004
E/A Ratio	0.69 (0.60, 0.80)	0.69 (0.58, 0.81)	0.789	0.71 (0.59, 0.83)	0.66 (0.59, 0.81)	0.287	0.71 (0.63, 0.80)	0.69 (0.59, 0.81)	0.348
Mitral Annular Early Diastolic Velocity (cm/s)	5.70 (4.70, 7.00)	4.50 (3.80, 5.30)	<0.001	5.81 (4.90, 6.80)	4.27 (3.60, 4.90)	<0.001	5.43 (4.74, 6.49)	4.32 (3.46, 5.14)	<0.001
E/e’ Ratio	11.50 (9.29, 13.92)	15.04 (13.48, 16.50)	<0.001	11.99 (9.46, 14.22)	15.02 (13.80, 17.00)	<0.001	13.00 (10.70, 14.50)	15.50 (14.00, 19.10)	<0.001

**Table 2:** Multivariate logistic regression analysis of independent predictors for spinal anesthesia-induced hypotension.

Variable	β	SE	Z Value	OR	95% CI	*p* Value
Age (y)	0.080	0.020	4.005	1.083	1.042, 1.127	<0.001
Body Mass Index (kg/m^2^)	0.099	0.035	2.826	1.104	1.031, 1.182	0.005
Dose of Bupivacaine (mg)	0.195	0.055	3.565	1.216	1.092, 1.354	<0.001
Sensory Block Level	−0.344	0.090	−3.809	0.709	0.594, 0.846	<0.001
Baseline Systolic Blood Pressure (mmHg)	0.031	0.008	3.877	1.031	1.015, 1.047	<0.001
History of Hypertension	0.579	0.233	2.481			0.013
Yes				1.783	1.129, 2.820	
No				-	-	
Interventricular Septum Thickness at End-Diastole (mm)	0.190	0.090	2.106	1.210	1.013, 1.444	0.035
e’ Mitral Annular Early Diastolic Velocity (cm/s)	−0.273	0.097	−2.816	0.761	0.630, 0.920	0.005
E/e’ Ratio	0.254	0.044	5.741	1.289	1.182, 1.405	<0.001

## Data Availability

The data presented in this study are not publicly available due to privacy and ethical restrictions. Anonymized data may be available from the corresponding author upon reasonable request, subject to institutional and data protection regulations.

## Supplementary Materials

The following supporting information can be downloaded at https://www.mdpi.com/article/10.3390/diagnostics16040557/s1, Table S1: Baseline characteristics of the training and internal validation sets.

## Abbreviations

SA	Spinal Anesthesia
SAIH	Spinal Anesthesia-Induced Hypotension
dIVCmax	Maximal Inferior Vena Cava Diameter
IVCCI	Inferior Vena Cava Collapsibility Index
ASA	American Society of Anesthesiologists Physical Status Classification System
ACEI	Angiotensin-Converting Enzyme Inhibitors
ARBs	Angiotensin II Receptor Blockers
BP	Blood Pressure
MAP	Mean Arterial Pressure
HR	Heart Rate
LAD	Left Atrial Diameter
IVSd	Interventricular Septum Thickness at End-Diastole
LVESD	Left Ventricular End-Systolic Diameter
LVEDD	Left Ventricular End-Diastolic Diameter
LVPWD	Left Ventricular Posterior Wall Thickness at End-Diastole
LVM	Left Ventricular Mass
LVEDV	Left Ventricular End-diastolic Volume
LVESV	Left Ventricular End-systolic Volume
E	Mitral Early Diastolic Peak Velocity
A	Mitral Late Diastolic Peak Velocity
e’	Early Diastolic Mitral Annular Velocity
E/A	E/A Ratio
E/e’	E/e’ Ratio
BMI	Body Mass Index
10 EPV	10 Events Per Variable
SD	Standard Deviation
LASSO	Least Absolute Shrinkage and Selection Operator
ROC	Receiver Operating Characteristic
AUC	Area Under the Curve
DCA	Decision Curve Analysis
CO	Cardiac Output
SVR	Systemic Vascular Resistance
CSF	Cerebrospinal Fluid
TTE	Transthoracic Echocardiography
LVDD	Left Ventricular Diastolic Dysfunction
