# Supplementary material for: A Nomogram for Predicting the Risk of Spinal Anesthesia-Induced Hypotension in Older Patients
Source: Diagnostics (Basel). 2026 Feb 13;16(4):557. doi: 10.3390/diagnostics16040557 (PMC12940050; doi:10.3390/diagnostics16040557)
Supplement: Supplementary file 1 [file diagnostics-16-00557-s001.zip › diagnostics-4096476-supplementary.pdf]

Table S1. Baseline characteristics of the training and internal validation sets.

Characteristic	Overall, N = 865	Training Set, N = 605	Internal Test Set, N = 260	P
**Sex**				0.236
**Male**	264 (30.5%)	192 (31.7%)	72 (27.7%)	
**Female**	601 (69.5%)	413 (68.3%)	188 (72.3%)	
**Age(y)**	72.0 (69.0, 76.0)	72.0 (69.0, 76.0)	72.0 (69.0, 77.0)	0.815
**ASA Physical Status Classification**				0.397
**II**	603 (69.7%)	427 (70.6%)	176 (67.7%)	
**III**	262 (30.3%)	178 (29.4%)	84 (32.3%)	
**Height (cm)**	160 (157, 167)	161 (157, 167)	160 (156, 167)	0.282
**Weight (kg)**	66 (60, 73)	67 (60, 75)	66 (60, 72)	0.512
**Body Mass Index (kg/m²)**	25.3 (23.0, 27.7)	25.4 (23.0, 27.8)	25.2 (23.2, 27.6)	0.802
**Type of Surgery**				0.087
**Orthopedics**	616 (71.2%)	424 (70.1%)	192 (73.8%)	
**Gynecology**	114 (13.2%)	76 (12.6%)	38 (14.6%)	
**Urology**	135 (15.6%)	105 (17.4%)	30 (11.5%)	
**Fasting Duration (h)**	13.10 (9.50, 15.52)	13.18 (9.50, 15.52)	13.03 (9.50, 15.52)	0.960
**Preoperative Fluid Administration (mL)**	138 (0, 440)	148 (0, 440)	129 (0, 440)	0.892
**Dose of Bupivacaine (mg)**	12.00 (10.50, 15.00)	12.00 (10.50, 15.00)	12.00 (10.50, 13.50)	0.282
**Sensory Block Level**	8.00 (8.00, 9.00)	8.00 (8.00, 9.00)	8.00 (8.00, 10.00)	0.156
**Baseline Systolic Blood Pressure (mmHg)**	144 (133, 157)	145 (134, 157)	143 (132, 155)	0.234
**Baseline Diastolic Blood Pressure (mmHg)**	75 ± 10	75 ± 10	76 ± 10	0.763
**Baseline Mean Arterial Pressure (mmHg)**	99 ± 10	99 ± 10	98 ± 10	0.700
**Baseline Heart Rate (bpm)**	74 (67, 82)	74 (66, 82)	74 (68, 82)	0.957
**Post-Spinal Anesthesia Systolic Blood Pressure (mmHg)**	113 (99, 128)	113 (100, 127)	113 (99, 129)	0.787
**Post-Spinal Anesthesia Diastolic Blood Pressure (mmHg)**	64 (56, 72)	64 (56, 72)	64 (56, 72)	0.673
**Post-Spinal Anesthesia Mean Arterial Pressure (mmHg)**	81 (73, 90)	81 (73, 90)	80 (72, 90)	0.835
**Post-Spinal Anesthesia Heart Rate (bpm)**	63 (56, 71)	63 (56, 72)	62 (56, 70)	0.513
**History of Hypertension**				0.817
**Yes**	451 (52.1%)	317 (52.4%)	134 (51.5%)	
**No**	414 (47.9%)	288 (47.6%)	126 (48.5%)	
**History of Diabetes Mellitus**				0.376
**Yes**	186 (21.5%)	135 (22.3%)	51 (19.6%)	
**No**	679 (78.5%)	470 (77.7%)	209 (80.4%)	
**History of Coronary Artery Disease**				0.263
**Yes**	117 (13.5%)	87 (14.4%)	30 (11.5%)	
**No**	748 (86.5%)	518 (85.6%)	230 (88.5%)	

**Left Atrial Anterior–Posterior Diameter (cm)**	3.60 (3.30, 3.90)	3.60 (3.30, 3.90)	3.60 (3.30, 4.00)	0.996
**Interventricular Septum Thickness at End-Diastole (mm)**	8.80 (8.00, 9.70)	8.80 (8.00, 9.70)	8.65 (8.00, 9.70)	0.513
**Left Ventricular End-Diastolic Diameter (cm)**	4.80 (4.50, 5.00)	4.80 (4.50, 5.10)	4.70 (4.50, 5.00)	0.245
**Left Ventricular End-Systolic Diameter (cm)**	2.90 (2.70, 3.20)	3.00 (2.70, 3.20)	2.90 (2.70, 3.20)	0.269
**Left Ventricular Posterior Wall Thickness at End-Diastole (cm)**	0.87 (0.80, 0.94)	0.87 (0.79, 0.94)	0.87 (0.80, 0.94)	0.906
**Left Ventricular Mass (g)**	142 (122, 163)	143 (121, 164)	139 (122, 159)	0.315
**Left Ventricular Ejection Fraction (%)**	68.0 (64.0, 71.7)	67.8 (63.8, 71.7)	68.5 (64.8, 72.0)	0.229
**Left Ventricular End-Diastolic Volume (mL)**	107 (92, 121)	107 (93, 122)	104 (91, 119)	0.263
**Left Ventricular End-Systolic Volume (mL)**	34 (27, 40)	34 (28, 40)	33 (26, 40)	0.212
**Mitral Early Diastolic Peak Velocity (cm/s)**	66 (56, 79)	66 (56, 79)	65 (55, 82)	0.734
**Mitral Late Diastolic Peak Velocity (cm/s)**	94 (84, 107)	94 (84, 107)	94 (84, 107)	0.833
**E/A Ratio**	0.69 (0.59, 0.81)	0.69 (0.60, 0.80)	0.68 (0.59, 0.82)	>0.999
**Mitral Annular Early Diastolic Velocity (cm/s)**	5.10 (4.18, 6.20)	5.10 (4.19, 6.20)	5.14 (4.10, 6.22)	0.869
**E/e' Ratio**	13.6 (10.9, 15.4)	13.4 (10.7, 15.4)	13.8 (11.1, 15.4)	0.531
